# No specimen left behind: industrial scale digitization of natural history collections

**DOI:** 10.3897/zookeys.209.3178

**Published:** 2012-07-20

**Authors:** Vladimir Blagoderov, Ian J. Kitching, Laurence Livermore, Thomas J. Simonsen, Vincent S. Smith

**Affiliations:** 1 Department of Life Sciences, Natural History Museum, Cromwell Road, London, SW7 5BD, UK

**Keywords:** Digitization, imaging, specimen metadata, natural history collections, biodiversity informatics

## Abstract

Traditional approaches for digitizing natural history collections, which include both imaging and metadata capture, are both labour- and time-intensive. Mass-digitization can only be completed if the resource-intensive steps, such as specimen selection and databasing of associated information, are minimized. Digitization of larger collections should employ an “industrial” approach, using the principles of automation and crowd sourcing, with minimal initial metadata collection including a mandatory persistent identifier. A new workflow for the mass-digitization of natural history museum collections based on these principles, and using SatScan® tray scanning system, is described.

## Introduction

Natural history collections are of immense scientific and cultural importance. Specimens in public museums and herbaria and their associated data represent a potentially vast repository of information on biodiversity, ecosystems and natural resources for the widest range of stakeholders, from governments and NGOs to schools and private individuals. Numerous examples of the uses to which biodiversity data derived from natural history collections have been put in research on evolution and genetics, nature conservation and resource management, public health and safety, and education are widely available (summarized in Chapman 2005, Baird 2010). The universe of natural history collection data has been estimated to be between 1.2 and 2.1 × 10^9^ units (specimens, lots and collections) (Ariño 2010). To ensure efficient access, dissemination and exploitation of such an immense wealth of biodiversity relevant data, it is evident that a well-coordinated and streamlined approach to global digitization is required, in particular because it is absolutely essential for the scientific value of the generated data that the outputs (images, metadata, etc.) are linked together and also back to the original specimens via unique identifiers (uIDs).

In recent years, substantial efforts and resources have been invested into the digitization of natural history collections, with museums and herbaria routinely employing specimen level collection databases to replace older, paper-based card indexes and ledgers. In theory, this should make dissemination of specimen data through biodiversity informatics portals such as the Global Biodiversity Information Facility (GBIF; http://www.gbif.org/) very simple and straightforward. However, the truth is that natural history collections are almost as far from complete digitization as they were 20 years ago. Ariño (2010) estimated that no more than 3% of biological specimen data is web-accessible through GBIF, the largest source of biodiversity information. Consequently, there is neither a central database of collection holdings, nor a complete collection index available to users. The reason for this deficiency is partly the immense effort it would take to digitize the vast number of collections units involved (Vollmar et al. 2010). The cost of traditional digitization workflows is vast, both in financial and human terms. Our simple calculations have shown that complete databasing of the ~30 million insect specimens housed in the entomological collection of the Natural History Museum, London, would require 23 years of continuous work from the entire departmental staff to complete (65 people). Depending on the particular collections and curatorial practices used, estimates vary from US$0.50 to several dollars per specimen to capture full label data (Heidorn 2011). The cost of traditional imaging and databasing of every natural history object in all European museums was recently estimated as €73.44 per object (Poole 2010). Thus, the complete digitization of all natural history collections may cost as much as €150,000 million, and take as long as 1,500 years.

The most common solution proposed to overcome the enormous cost of digitization is prioritization based on user demand (Berents et al. 2010). Currently, most digitization projects concentrate their efforts on obtaining high quality images of selected specimens accompanied by high quality data (e.g., comprehensive and expertly interpreted label information) rather than total collections coverage. Such specimen-centric digitization efforts are thus inevitably fragmented into numerous small-scale and labour-intensive projects that usually image single specimens, one at a time.

To solve the problem of cost, as well as the inherent fragmentation in collection based biodiversity informatics, new, industrial-scale approaches to digitization are clearly needed. The larger a digitization project becomes, the lower are the transaction costs and thus the lower is the cost per specimen. Such an industrial-scale process must necessarily fulfil certain standardized criteria if it is to be of use to and adopted by a wide spectrum of natural history collections:

– As much as possible of the procedure must be automated, except when physical handling of specimens is necessary.

– The approach should, whenever possible, focus on “wall-to-wall” total digitization of entire collections, because it is faster to digitize an entire collection than to select individual specimens or drawers of particular interest.

– Complicated labour-intensive procedures must be divided into a series of separate, shorter steps, each with a distinct outcome. For example, preparation of specimens for imaging should be a separate step from the imaging itself; and unique specimen identifiers can be assigned simultaneously to all specimens in a drawer rather than individually and sequentially. Such a modularised process can then be more easily crowd-sourced among the professional and volunteer communities. Properly organized crowd-sourcing projects would be able to mobilise the efforts of thousands of enthusiasts around the world (Hill et al. 2012).

– Collection of metadata must be simplified and standardized. In most cases, digital representation of the specimen and minimal metadata (uID, specimen location in the collection) is sufficient for collection management purposes. Only minimal information should be collected when initially digitizing an entire collection, but in such a way that it can be amended and expanded upon later.

Here we describe a new method for “wall-to-wall” mass-digitization of natural history museum collections based on the SatScan® tray scanning system. The method allows for standardized scanning of museum collection trays of the highest image quality possible, followed by simplified (and easily expandable) collection of metadata.

## Methods

**Figure 1.**
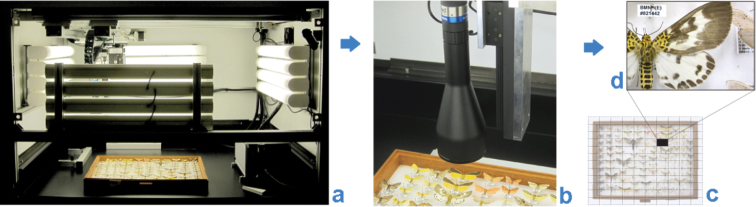
SatScan imaging: **a** SatScan machine **b** specimens being imaged **c** individual frames aligned **d** fragment of a stitched image; final resolution of the stitched image ~11 lines/mm.

The Natural History Museum (NHM), London, has been working with SmartDrive Limited (http://www.smartdrive.co.uk/) since 2009 on the development of one of the company’s products, the SatScan® collection scanner (Fig. 1). From this collaboration, we have developed a workflow that we consider meets our needs for the industrial-scale digitization of a significant part of the NHM’s collections. The system is particularly suited to the digitization of multiple, uniformly mounted or laid out specimens, such as pinned insects and smaller geological or mineralogical objects in standardized collection drawers, horizontally-stored microscope slides and herbarium sheets.

**Figure 2.**
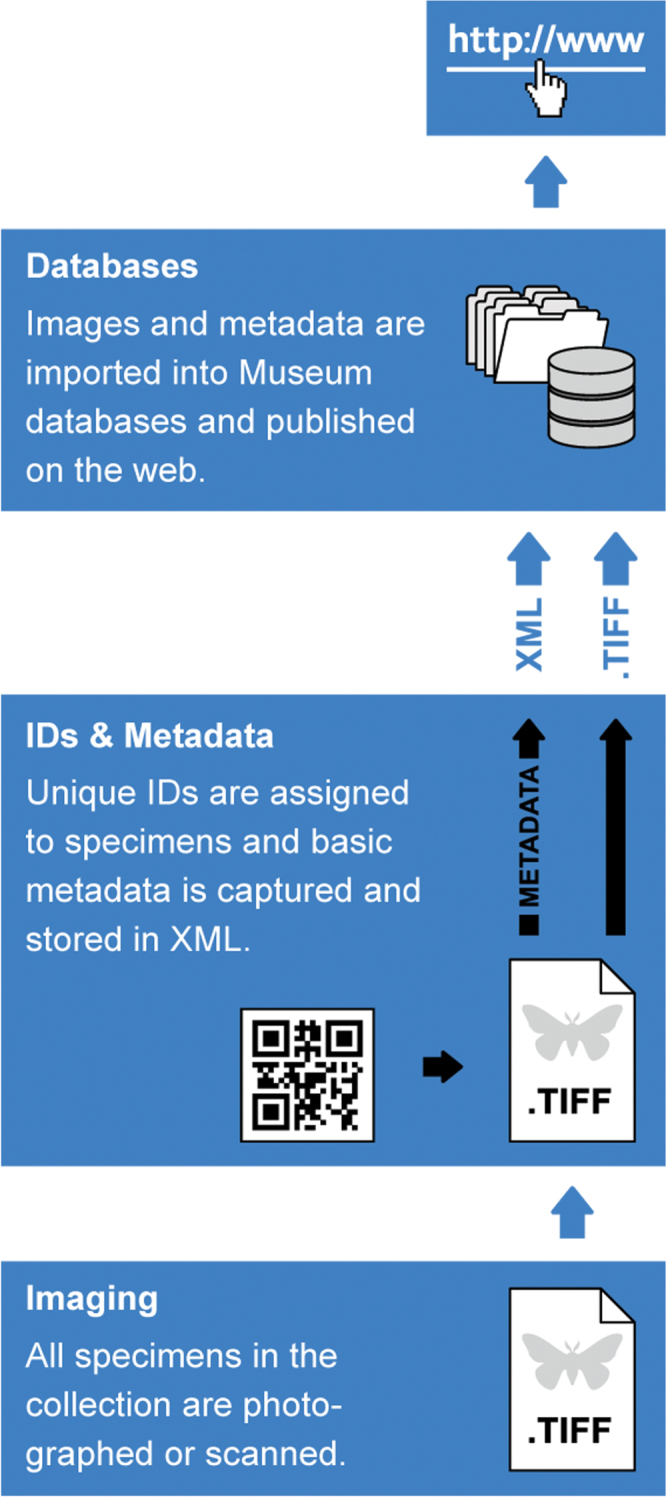
Image based digitization workflow consisting of four stages: Imaging, Metadata capture, Institutional databading and Publication.

The digitization workflow envisioned for the NHM (Fig. 2) comprises three steps:

### Imaging

The SatScan® collection scanner is capable of producing high-resolution images of entire collection drawers (see Table 1, Blagoderov et al. 2010, Mantle et al. 2012). The specific configuration of the system has changed somewhat from that described in the report, such that now a USB CMOS UEye-SE camera (model # UI-1480SE-C-HQ, 2560×1920 resolution) is used in combination with Edmund Optics telecentric TML lenses of 0.3× (#58428) and 0.16× TML (#56675). A camera with attached lens is moved in two dimensions along precision-engineered rails positioned above the object to be imaged. A combination of hardware and software provides automated capture of high resolution images of small regions of interest, which are then assembled (“stitched”) into a larger panoramic image, generating the final image of the entire drawer. This method maximizes depth of field of the captured images and minimizes distortion and parallax artefacts. Analogous solutions for large-area imaging which have been developed independently include GigaPan (Bertone et al. 2012), MicroGigaPan (Longson et al. 2010) and DScan (Schmidt et al. 2012).

**Table 1.** Resolution and depth of field of the system as compared with a Canon EOS450D DSLR camera using a Canon MP E-65 macrolens (USAF: the smallest resolvable element on 1951 US Air Force resolution test chart; MRD: minimal resolved distance, size of the smallest visible object on image)

Objective	Sensor Resolution	Aperture	Depth of Field, mm	Resolution		
				USAF	Lines/mm	MRD, μm
SatScan 0.16× lens	1280×960	Open	5	3–4	11.3	44
		Dot	10	3–4	11.3	44
		Closed	>70	2–5	6.35	79
	2560×1920	Open	5	4–3	20.16	25
		Dot	14	4–1	16.0	31
		Closed	>70	3–2	8.89	56
SatScan 0.3× lens	1280×960	Open	2.5	4–2	17.95	28
		Dot	4.5	4–2	17.95	28
		Closed	30	3–4	11.3	44
	2560×1920	Open	1.5	5–3	40.3	12
		Dot	3	5–2	36.0	14
		Closed	35	3–5	12.7	39
Canon MP-E65 lens, 1×	4272×2848	2.8	0.5	5–6	57	8.8
		16	4	-	-	-
Canon MP-E65 lens, 5×	4272×2848	2.8	<0.3	8–1	256	2
		16	2	6–2	71.8	7

### Metadata capture

**Figure 3.**
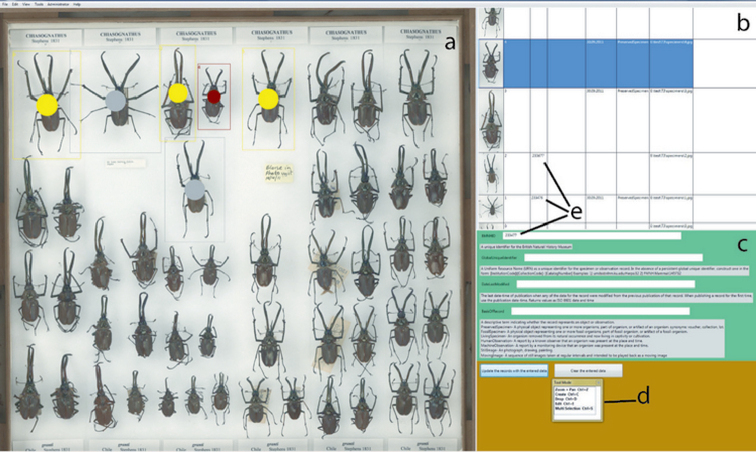
Metadata Creator software: **a–c** working areas **a** drawer image **b** specimen records **c** annotation fields **d** tool selector **e** unique IDs.

A prototype software program, Metadata Creator, has been designed to allow fast capture of specimen data and associating these with the image of the specimen (Fig. 3). Users can mark individual specimens on the panoramic image by drawing rectangular boxes around them, selecting these areas and annotating them individually or in batches. Methods for marking the specimen, editing regions of interest and selection of multiple specimens are analogous to those used in many common graphic applications and so will be familiar, even to inexperienced users.

Specimen metadata is captured in a series of fields that are compatible with the Darwin Core 1.4.1 schema (http://rs.tdwg.org/dwc/) and which can be customized to particular user requirements. To maximize throughput, only basic metadata are collected at this stage. These will generally include a unique collection number of every specimen (see below, barcodes), collection identification (to the available curatorial level, e.g. to species/subspecies for the “Main Collection” and family/order for unsorted accessions), and, if possible, biogeographic region/country. Taxon names are looked up from an index derived from the NHM Collections Management Database. A completed project comprises a folder with an archival image of the drawer, full-resolution images of individual specimens cut-out from the drawer image, and an XML file containing annotations and links to specimen images (Appendix 1). Trials have demonstrated that 10–20 seconds per specimen is required to capture basic metadata using the Metadata Creator Software. A unique ID for the drawer is also recorded. As the NHM Collection Management System already includes a complete collections index (a brief description of the content of every drawer), no additional information is required.

### Assigning uIDs

Every specimen is assigned a unique number under which it will be registered in the NHM Collections Management Database. It is a requirement of collections management procedures that a label bearing the specimen’s uID is attached to the specimen. To streamline this part of the process, it is subdivided into the following steps:

1. A sequence of unique numbers is generated from the NHM Collections Management Database.

2. Labels that include both a human-readable number and a machine-readable barcode are printed.

3. The operator labels the specimens by selecting a specimen on the drawer image, pinning a label under the specimen, and scanning the barcode, thereby adding the uID into the corresponding field of Metadata Creator. Barcodes can be pinned facing up or down depending on curatorial practice; the former has the advantage of visibility on the image. In this case imaging, of course, has to take place after assigning uIDs. Images of individual specimens for which the metadata have been collected and individual numbers assigned are automatically marked on the drawer image with a grey spot, allowing easy visualization of progress.

4. When all specimens have been labelled and recorded, the XML file and corresponding specimen images are imported into the NHM Collections Management Database.

We must emphasize that Metadata Creator is a prototype software application; much more development is needed for to perfect its functionality, user interface, and integration with the Museum’s information systems.

## Results

A preliminary assessment of the SatScan® system was undertaken and reported upon by Blagoderov et al. (2010). Based on their findings, a series of recommendations were made for improvements and possible longer term developments to the hardware, software, imaging system and ergonomics. An updated system was delivered to the NHM in September 2011 and further trials were then conducted. This newer version of SatScan® provides non-extrapolated resolution of the final images from 11.3 to 40.3 lines/mm and a minimum resolved distance of 79 to 12μm, depending on the lens and sensor settings employed (Table 1). The maximum depth of field has been increased slightly from 80mm to 85mm. Although focus stacking is implemented in the current version of the system, in most cases it is not necessary. For the majority of collections drawers, specimens are presented at a more-or-less uniform height and within the available depth of field; focus stacking is really only necessary for those drawers where specimens are pinned at markedly different heights or are particularly deep (e.g. fossils and mineralogical samples). The average time to scan a typical collection drawer without focus stacking is between four and six minutes, depending upon size (eight to ten minutes including logistics, Table 2). This generally translates to about two seconds per specimen. Thus, in a working day, an operator could image up to 70 drawers. These would then be stitched into the final images using an overnight batch process (see average figures in Table 2). The resulting images vary in size from 0.3 Gpx to 5 Gpx (10^9^ pixels; 250 MB – 3 GB compressed TIFF files) depending on the imaging area, lens and resolution used. However, use of the highest resolution in mass digitization projects may not always be practical. We did not conduct extensive tests with the highest resolution of camera/higher magnification of lens because a 64-bit version of software is needed to handle the stitching process for files of this size, and this was not available at the time of trials.

The part of the process that involves marking of specimens and metadata capture using Metadata Creator has not been as thoroughly tested and we have yet to trial the part of the procedure that produces barcode labels and attaches these to specimens. However, preliminary results involving mock elements indicate that it will take about ten seconds per specimen. This time will be extended for those specimens that already have a human-readable uID (a “BMNH(E)” number, for example) but no barcode label, because then the former will have to be manually entered into Metadata Creator and a new barcode label printed. However, relatively few NHM insect specimens (about 1.2%) have so far been databased and assigned a uID.

The entomology collections of the NHM have about 30 million insect specimens, mostly pinned, housed in 135,000 collections drawers. Assuming that 80% of the collection is appropriate to be imaged using the SatScan® system, rough calculations based on the above figures suggest that the entire collection could be imaged and basic metadata captured in 18 person-years.

**Table 2.** Scanning and stitching times for different types of drawers.

Drawer type	Number of drawers in trials	Dimensions, mm	Number of frames	Average scanning time (including logistics), min	Average stitching time, min	File size, Mb
Main collection and accessions	236	500×400 or 470×450	17×14 or 16×15	8.52	12.65±1.54	488.20±30.21
Rothschild and Rhopalocera	144	560x540 or 570x 555	21×17 or 22×17	10.13	25.41±4.21	715.90±89.58

## Discussion

Although images acquired through an industrial digitization process might be considered to be of limited use for taxonomic purposes, because they feature only one aspect of the specimens and may not contain necessary morphological details or label data, they could prove very useful for a variety of other purposes. Obvious collection management applications include improved collection audit and security, as well as improving accessibility of the collection. For research purposes, such acquired images could prove very valuable in morphometric analyses and phenological population studies. In addition, the public engagement aspect of industrial digitization activities should not be underestimated. Online public access to high resolution images and metadata will likely enhance public awareness of the importance of local and national collections (as well as engendering a sense of shared ownership). Moreover, high quality images will open up the possibility for fast and reliable automated or semi-automated specimen identification and thus encourage environmental “citizen-science”, such as recording distributional or abundance changes of key species.

Major problems remaining with the described approach are largely concerned with the time taken to scan specimens/samples and to collect metadata. Even with a simple approach, scanning a specimen takes approximately two to four seconds followed by 10–20s for annotation and/or barcoding. Furthermore, only basic metadata are collected under the scenario described above. Indeed, in the worst case, say a drawer of unidentified mixed organisms from several phyla, only a uID will be associated with each of the specimens. It may then be argued that this will compel museums and herbaria to create essentially incomplete records with which to populate their collection databases. However, such records are comparable to stub pages in Wikipedia, empty at the moment but capable of being filled and edited in due course. Indeed, there is a case to be made for the opposite viewpoint, that there is no point collecting complete metadata if these are not going to be used for any purpose. Finally, it should be noted that the industrial digitization process described above only works relatively seamlessly for more-or-less uniformly preserved and presented specimens, such pinned insects in drawers and herbarium sheets. It is unlikely to be satisfactory for pickled specimens in jars of ethanol. These collections may have to be digitized using a different protocol.

Approximately 90% of the time required for digitization is spent on capturing metadata and labelling specimens. While the latter involves physical handling of the specimens and must be performed by experienced staff, selection of specimens in the drawer images and annotation thereof can be undertaken in a virtual environment. In many cases, the basic information to be collected can be seen in the drawer image. Implementing an open source web application that duplicates the functions of Metadata Creator and publication of drawer images using algorithms involving a pyramid of tiles (produced using Zoomify™ (http://www.zoomify.com/) or Google Maps (http://maps.google.com/), for example) will allow volunteers from around the world to participate in digitization of the collection and will decrease the time needed to process a specimen by at least 50%.

The next step in facilitating the digitization process might be to undertake “virtual curation”. Here, uIDs are assigned to each specimen, records are created in the collection management database and corresponding specimen images linked to these records, but the specimens themselves are not labelled until it becomes necessary to handle the specimen physically for some other purpose (curation, loan, identification, dissection, etc.). Of course, these procedural changes would require a major cultural shift for Collections Management staff.

Revised, though still simplistic, calculations now show that the entire NHM collection of insects could be imaged in 12.88 person-years and completely digitized without crowd-sourcing in 118 person-years. Collecting basic information and attaching a barcode to a specimen would take approximately 10-30 seconds. Per-specimen cost under the current (2012) economic climate would thus be as low as £0.12. If we limit SatScan-based digitization to large and medium-size insects (up to 5 mm in length), the total time required is 58 man-years. This effort does not seem insuperable considering that the NHM insect collection is managed by 26 permanent curatorial staff, assisted by a number of people in short-term contracts and volunteers.

Despite the potential perceived drawbacks, image-based basic digitization can nevertheless mobilize hundreds of millions of biological specimens in a relatively short period of time. It is estimated that entomological specimens constitute up to 40% of all natural history specimens (Ariño 2010). Some palaeontological, zoological and mineralogical specimens, including microscopic slides, are also stored in collection drawers and trays that are amenable to simultaneous imaging. Thus, the majority of natural history specimens could potentially be digitized using industrial imaging.

The return on investment in total collection digitization will be enormous. It will open up collections to the world, facilitating their use, and help create a global collection index that can be used to set priorities for further digitization. Basic digitization of all the world’s holdings of insects (800 million specimens) could be completed in less than 4000 person-years. This may sound like a huge figure, but divided among approximately 1,300 collections and potentially tens of thousands of professionals and volunteers, the work could be completed much quicker, perhaps in only a few years. "Furthermore, emerging technologies in the near future will undoubtedly decrease time and costs, while increasing data quality. Complete image-based basic digitization of insect and plant collections would produce at least 30 Pb (10^15^ bytes) of data, which constitutes ~0.0006% of the current data hosted on the Internet. At £0.2 per specimen, the cost of digitizing 2,000 million natural history specimens may appear to be an eye-wateringly high figure of £400 million. However, divided among ~4,000 natural history collections, this reduces to an average project cost of £100,000, which is equivalent to the size of a relatively modest research grant. To this the cost of imaging equipment must be added. At present, a SatScan system costs between £25,000 and £60,000, depending on the options to be implemented and the service agreement chosen, but less expensive alternative solutions are also being developed (Bertone et al. 2012, Dietrich et al. 2012, Schmidt et al. 2012).

Regardless of the technology used, mass digitization will nevertheless follow the same general approach, which includes mechanisms that enrich digital media with specimen-level metadata. This enrichment will:

1. Facilitate open dissemination of data so that it can be discovered and accessed by stakeholders, reducing both the need for physical access to collections and the number of loans;

2. Enable large-scale manipulation and integration of collection data, supporting stakeholders in their monitoring and management of information on ecosystems, biodiversity and natural resources;

3. Enhance curatorial activities, allowing the condition of loans to be tracked and reduce identification inaccuracies;

4. Protect biodiversity heritage by reducing the need to handle irreplaceable specimens;

5. Improve collections security by providing base-line images against which damage and thefts can be monitored;

6. Support disaster management, such that should the worst happen to a collection, its digital representation will continue to provide a valuable resource;

7. Raise natural history collections profiles, resulting in improved resources for further research;

8. Contribute beyond the traditional remit of museums and herbaria into new areas of interest, particularly education and public understanding of science; and

9. Support biodiversity legislation and data repatriation, which is an increasing requirement under both the 1992 Convention on Biological Diversity and the subsequent 2010 Nagoya Protocol on Access and Benefit-sharing.

## Appendix 1

An example of XML output of Metadata Creator.

<?xml version=”1.0” encoding=”utf-8”?>

<Project xmlns:xsi=”http://www.w3.org/2001/XMLSchema-instance” xmlns:xsd=”http://www.w3.org/2001/XMLSchema”>

<Templates>

<key>BMNHID</key>

<value>A unique identifier for the British Natural History Museum</value>

<key>GlobalUniqueIdentifier</key>

<value>A Uniform Resource Name (URN) as a unique identifier for the specimen or observation record. In the absence of a persistent global unique identifier, construct one in the form: [InstitutionCode]:[CollectionCode]: [CatalogNumber] Examples: 1) urn:lsid:nhm.ku.edu:Herps:32 2) FMNH:Mammal:145732</value>

<key>DateLastModified</key>

<value>The last date-time of publication when any of the data for the record were modified from the previous publication of that record. When publishing a record for the first time, use the publication date-time. Returns values as ISO 8601 date and time</value>

<key>BasisOfRecord</key>

<value>A descriptive term indicating whether the record represents an object or observation.

</value>

</Templates>

<Specimens>

<Specimen>

<DarwinCoreData>

<key>ImageURL</key>

<value>E:\test\T3\specimens\G1_2_0000.jpg</value>

<key>BMNHID</key>

<value> </value>

<key>GlobalUniqueIdentifier</key>

<value> </value>

<key>DateLastModified</key>

<value> </value>

<key>BasisOfRecord</key>

<value>preserved specimen</value>

</DarwinCoreData>

<SpecimenIndex>0</SpecimenIndex>

<ImageDimensions>

<Left>519.635599159075</Left>

<Top>1490.562857142857</Top>

<Width>2247.9887876664334</Width>

<Height>3511.8564285714283</Height>

</ImageDimensions>

</Specimen>

<Specimen>

…………………………………………………………………………………………………………………………………………………………………………………………………………………………………..

</Specimen>

</Specimens>

</Project>
